# Assessing Current Interest, Knowledge, and Inquiries About Vasectomies in Urology Patients and Medical Students

**DOI:** 10.7759/cureus.47074

**Published:** 2023-10-15

**Authors:** Rachel Alef, Harvey N Mayrovitz

**Affiliations:** 1 Osteopathic Medicine, Nova Southeastern University Dr. Kiran C. Patel College of Osteopathic Medicine, Davie, USA; 2 Medical Education, Nova Southeastern University Dr. Kiran C. Patel College of Allopathic Medicine, Davie, USA

**Keywords:** roe v. wade, abortion, medical students, urology patients, vasectomy knowledge, vasectomy interest

## Abstract

Background and objective

Since the overturning of Roe v. Wade, there has been an increased interest in vasectomy procedures. This study aims to analyze interest, knowledge, and inquiries about the vasectomy procedure among urology patients and osteopathic medical students since this overturn. It also seeks to determine if this data varies between the ages and sexes of participants. It is hoped that the findings will aid in the development of a standardized educational plan that might be provided to urologists for future use with patients and their partners.

Methods

Surveys consisting of 10 questions regarding interests, knowledge, and inquiries about vasectomy procedures and the changes in interest following the Roe v. Wade decision were distributed to urology patients and osteopathic medical students.

Results

Female students had an increased interest in their current or future male partner obtaining a vasectomy procedure compared to a year ago, whereas older urology patients and male students did not. Based on the responses to the posed queries, the most important information to include in a standardized educational plan for patients is the overall risks and their likelihood, the likelihood of reversal, and the procedure's recovery time and surgical details.

Conclusion

Female students' increased interest in the vasectomy procedure may be due to the recent overturn of Roe v. Wade. Therefore, physicians must counsel their male patients’ female partners or interested females appropriately regarding vasectomies, as more may now be interested. Additionally, an educational plan based on this study’s data may be utilized with future urology patients. Placing emphasis on what patients want to know may help ease patient’s associated anxiety with their future procedure and strengthen the relationship between the patient and physician.

## Introduction

Since the overturning of Roe v. Wade, there have been some reports of an increase in vasectomy interest across the United States. Bole et al. released an early paper noting an increase in vasectomy procedures in 2023 [1]. In that study, vasectomy data from a healthcare system in the Midwest United States was collected before and after the overturning of Roe v. Wade. Before the overturning (BTO), 58.6% of 116 men who had an initial consultation had a vasectomy procedure. After the overturning (ATO), 66.2% of 142 men who had an initial consultation had a vasectomy procedure. Although these overall percentages were not shown to be statistically significant (p = 0.21), there was a difference for men less than 30 years old. For this sub-group, 10.3% had a vasectomy BTO, whereas 23.9% had a vasectomy ATO (p = 0.005). Additionally, analyzing between 2018 and 2022, it was found that there was a significant increase in the median number of vasectomy procedures per month ATO (104 prior v. 218 post; p = 0.03) [1]. Another study found higher “vasectomy” search rates two weeks ATO to be 47.3% compared to “vasectomy” searches two weeks before the overturn at 17.2% (p < 0.05) [2]. A third study analyzing Google trends of “vasectomy” noted a 3.6 times increase in searches the week after the Roe v. Wade decision compared to its prior search history [3]. According to a national database collected by Zhang et al., there is also a notable increase in the actual procedure since the overturn [4]. 

Although the prior indicators suggest a greater general interest in vasectomies ATO, it is unclear whether this applies to actual current urology patients and to younger persons such as those enrolled in medical school who would be expected to have a greater knowledge of the vasectomy process. One goal of this study was to investigate this issue by comparing view changes from BTO to ATO of male and female subjects with varying ages and to assess their knowledge of the vasectomy process. An additional goal was to create set educational guidelines based on collected data for urologists to provide to patients’ pre-vasectomy procedures. The American Urological Association lists 11 pre-operative topics that should be discussed with patients prior to receiving a vasectomy procedure [5]. However, there is limited research regarding current patient knowledge about vasectomies in America and what patients deem the most important information. Additionally, according to a study conducted by Kianian et al., online resources regarding the vasectomy procedure were found to be difficult to understand for the general population. Utilizing indices such as Simple Measure of Gobbledygook (SMOG) and Gunning Fog (English readability tools), it was found that the average online information regarding vasectomies was written at an undergraduate reading level [6]. According to the American Medical Association, health information should be written at a fifth or sixth-grade reading level, to make sure instructions and details are inclusive of all people [7]. Therefore, interested patients may already be faced with a disadvantage when attempting to learn about the procedure on their own. It would be helpful for urologists to place educational emphasis on topics that patients deem to be the most important. 

## Materials and methods

Participants

There were two groups of participants: one group recruited directly from two urology offices (patients) and one from an email solicitation of osteopathic medical students (students). Both groups completed a 10-question survey designed to elicit their feelings on a variety of aspects related to their current views of vasectomy vs. their views one year ago, which would have been prior to the Roe v. Wade decision of 2022. For patients, surveys were handed to them by the front desk staff when they checked in for their appointments. For students, the survey was housed in Research Electronic Data Capture (REDCap) which is a secure web application, hosted by NSU KPCOM [8,9]. A link to that survey was emailed to all enrolled osteopathic medical students at NSU-KPCOM (first, second, third, and fourth-year students) at both Florida campuses (Davie and Clearwater). The study was approved by the Nova Southeastern University Institutional Review Board (IRB Study No. 2023-10 and 2023-98).

Surveys

Patient surveys were paper copies of the 10 questions that were multiple-choice and divided into four main categories. It started with demographic questions asking patients for age, and if they were male, a female with a current male partner, or a female without a current male partner. The survey then asked patients about comparative vasectomy interest: if, compared to a year ago, their personal interest (or interest in current or possible future male partner) in having a vasectomy has changed. The third category asked about current patient knowledge of vasectomies (knowledge of the procedure, associated risk factors, and what they believe is the chance of successfully reversing a vasectomy). Last, the survey asked patients what would be the most and second most important thing to know regarding vasectomies; patients were given the following options: preparing for the procedure, details of the surgical procedure, amount of pain associated with the procedure, risk factors and their likelihood, recovery timetable for the procedure, and chances of reversal of a vasectomy. There was an additional optional question for patients who had any comments they’d like to provide. The survey questions are in Table 1.

**Table 1:** Survey questions and response options for patients

Survey Questions	Respondent Selection Choices
What is your age?	18-25
	26-35
	36-45
	46-55
	56-65
	Over 65
Which one of the following applies to you?	I am a female who has a current male partner
	I am a female but don’t have a current male partner
	I am a male
If you are a MALE please answer this question, otherwise skip to #4. Compared to a year ago, how much has your interest in having a vasectomy changed?	Interest increased a lot
	Interest increased a little
	Interest unchanged from a year ago
	Less interested
	Not relevant since I already had a vasectomy
If you are a FEMALE please answer this question, otherwise skip to #5. Compared to a year ago, how much has your interest in your current or a possible future male partner having a vasectomy changed?	Interest increased a lot
	Interest increased a little
	Interest unchanged from a year ago
	Less interested
	Not relevant since my partner already had a vasectomy
How much do you know about the actual vasectomy procedure?	A lot
	A little
	Essentially nothing
How much do you know about the risk factors associated with a vasectomy?	A lot
	A little
	Essentially nothing
What do you think is the chance of successfully reversing a vasectomy?	0-10%
	30-50%
	70-85%
	90-100%
Which of the following aspects of a vasectomy would be MOST important to you to learn more about	Preparing for the procedure
	Details of the surgical procedure
	Amount of pain associated with the procedure
	Risk factors and their likelihood
	Recovery timetable for the procedure
	Chances of reversal of a vasectomy
Which of the following aspects of a vasectomy would be the 2ND MOST important to you to learn more about?	Preparing for the procedure
	Details of the surgical procedure
	Amount of pain associated with the procedure
	Risk factors and their likelihood
	Recovery timetable for the procedure
	Chances of reversal of a vasectomy
Please optionally provide any comments you want related to the issue of vasectomy	Patients are free to respond however they’d like.

The survey for students was the same as for patients, except it asked students about their current year in medical school, and the optional question for any additional comments was not provided in this survey. The survey questions are in Table 2.

**Table 2:** Survey questions and response options for students

Survey Questions	Respondent Selection Choices
What is your age?	18-23
	24-29
	30-35
	36-41
	42 or older
Which of the following applies to you?	I am a female who has a current male partner
	I am a female who doesn’t have a current male partner
	I am a male
Which best describes your current medical school status?	M1
	M2
	M3
	M4
If you are a MALE, please answer this question, otherwise go to question #5. Compared to a year ago, how much has your interest in getting a vasectomy changed?	Increased a lot
	Increased a little
	Interest unchanged from a year ago
	Less interested
	Never had any interest
If you are a FEMALE, please answer this question, otherwise go to question #6. Compared to a year ago, how much has your interest in your current or a possible future male partner getting a vasectomy changed?	Increased a lot
	Increased a little
	Interest unchanged from a year ago
	Less interested
	No interest since my partner already had a vasectomy
How much do you know about the actual vasectomy procedure?	A lot
	A little
	Essentially nothing
How much do you know about the risk factors associated with a vasectomy?	A lot
	A little
	Essentially nothing
What do you guess is the chance of successfully reversing a vasectomy?	0-10%
	30-50%
	70-85%
	90-100%
Assuming that there were to be a future vasectomy, which of the following aspects of a vasectomy would be MOST important to you to learn more about?	Preparing for the procedure
	Details of the surgical procedure
	Amount of pain associated with the procedure
	Risk factors and their likelihood
	Recovery timetable for the procedure
	Chances of reversal of a vasectomy
Assuming that there were to be a future vasectomy, which of the following aspects of a vasectomy would be 2ND MOST important to you to learn more about?	Preparing for the procedure
	Details of the surgical procedure
	Amount of pain associated with the procedure
	Risk factors and their likelihood
	Recovery timetable for the procedure
	Chances of reversal of a vasectomy

Data collection

For patients, data forms in sealed envelopes were collected from the front desk staff and kept in a labeled container behind the desk. The investigators collected the envelopes at least twice monthly and entered the data in a Google form. For students, data was collected via REDCap survey collector. There were 76 responses from patients and 106 responses from students, and all were included for analysis. Data from both groups was compiled in a single SPSS file for analysis.

Analysis

Data are presented as counts of responses for each query and for patients and students separately. The appropriate comparisons between responses for a given query between genders were done using chi-square analyses with a p-value of 0.01 taken to indicate a statistically significant difference. 

## Results

Numbers and age ranges

There were 76 patient respondents of which 62 (82%) were male. Of the 14 female respondents, nine (64%) had a male partner and 5 (36%) did not. Overall, 84% of respondents were over the age of 45, and 37% were over the age of 65. There were 106 student respondents of which 44 (41.5%) were male. Of the 62 female respondents, 25 (40.3%) had a current male partner and 37 (59.7%) did not. Of the student respondents, 77.4% were aged between 24 and 29 years, and 17.9% were aged between 18 and 23 years. Most of them were first-year (38.7%) or second-year students (32.1%), and the remaining were third and fourth-year students (29.3%).

Change in interest in vasectomy

**Figure 1:**
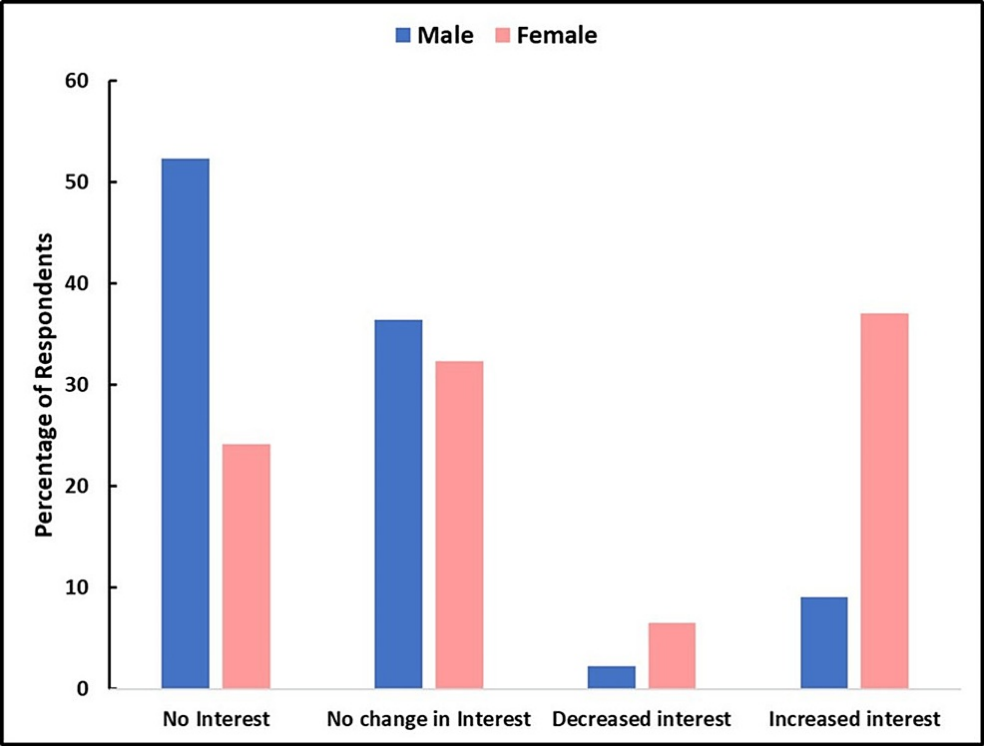
Medical student comparisons of vasectomy interest by gender

For patients, 54 (87.1%) of males responded to this query. Of those, 12 (22.2%) already had had a vasectomy. For those who had not (42, 77.8%), there was no change in vasectomy interest in 16 (38.1%), less interest in 18 (42.8%), and more interest only in 8 (19.1%). Of 14 female patient respondents, only eight (57.1%) answered this query. Of those respondents, two (25%) stated it was not relevant to them. Out of the remaining, three (37.5%) indicated less interest, and three (37.5%) indicated increased interest. For students, 100% of males and females responded to this query and no males had had a vasectomy. Of the 44 male students, 23 (52.3%) stated they never had any interest in a vasectomy, 16 (36.3%) had no change in their interest from one year ago, and only four (9.1%) stated that their interest increased a little or a lot. Contrastingly, for the 62 female students, 15 (24.2%) stated they never had an interest in a vasectomy for a partner, 20 (32.3%) indicated no change in their interest from a year ago, and four (9%) stated they had increased interest. Thus, a total of 39 female students either had no interest, no change in interest, or had less interest. However, 23 (37.1%) stated that their interest had increased a little or a lot from a year ago. Comparing these gender responses via chi-square analysis yielded a chi-square value of 10.63, corresponding to a p-value of 0.001. Thus, there was a statistically significant difference in the views of males and females regarding this query. Figure 1 graphically summarizes the main results regarding student gender differences in vasectomy interest.

Knowledge of the vasectomy procedure and risk

For patients, 72 (94.7%) responded to this query with two males and two females being non-responders. Of the responders, 40% knew essentially nothing about the vasectomy procedure, 28% knew a little, and 32% knew a lot about it. Specifically with respect to knowledge of the risk of a vasectomy, 56% knew essentially nothing, 21% knew only a little, and 24% stated they knew a lot. For students, 9.4% knew essentially nothing about the vasectomy procedure, whereas 71.7% had little knowledge, and 18.9% knew a lot. With respect to knowledge of risk, 29.2% knew essentially nothing, 55.7% knew a little, and 15.1% knew a lot about the risk.

Most important things to learn about a vasectomy

For this query, 54 patients (71.0%) responded. Of the responders, 21 (38.9%) selected the most important vasectomy-related topic to learn about was the overall risks of the procedure. Overall choices are summarized in Table 3.

**Table 3:** Most important vasectomy topics respondents wanted to learn about Entries are the percentage of respondents selecting the feature of concern.

	Patients (Urology Office Patients)		Students (Medical Students)	
Feature of concern	Most Important	2nd Most Important	Most Important	2nd Most Important
Risks and their likelihood	38.9	11.1	55.7	27.4
Vasectomy reversal	16.7	25.9	28.3	20.8
Recovery time	16.7	20.4	3.8	13.2
Surgical details	14.8	24.1	5.7	19.8
Pain	9.3	7.4	5.7	16.0
Preparing	3.7	11.1	0.9	2.8

The second most important vasectomy-related topic the patients wanted to learn about was the likelihood of reversal, selected by 14 patients (25.9%), but closely behind was the desire to learn more about the surgical details of the procedure (24.1%). For the 63 (82.9%) respondents that guessed as to the success of a vasectomy being reversible, there was a wide range, from 30.2% guessing a reversal rate of 0-10% to 19% guessing a 90-100% reversal rate. For students, the most important vasectomy-related topic to learn about was also the overall risks of the procedure, but compared to patients, there was a higher percentage (55.7%) of students indicating this. The second most important vasectomy-related topic that students wanted to learn about was also the risks and their likelihood (27.4%). For all students, their guesses as to a vasectomy being reversible varied, with 5.7% guessing between 0% and 10%, whereas 62.3% guessed a reversal rate between 70% and 100%.

## Discussion

The survey indicated that patients did not have much of an increase in vasectomy interest compared to a year ago, and in fact, most had less interest in the procedure. According to a study conducted by Naelitz et al., the average age of men obtaining a vasectomy post-COVID-19 pandemic was 39.6 years old compared to 38.5 years old prior to the pandemic (measured data using the electronic medical record at a medical center from 11/01/2011 to 10/31/2021) [10]. Most patients in this study were over the age of 45, and their older age may explain their lessened interest in the vasectomy procedure. There appeared to be a gender difference in vasectomy interest, since only 19% of males had an increased interest, compared to 33% of females who had an increased interest. Considering there were only 14 females in the patient study, it would be interesting for future studies to include a greater number of males and females to see if there was a statistically significant difference in interest between males and females. The trend does show females having more interest compared to males in the vasectomy procedure. Overall, since the overturning of Roe v. Wade, it seems as though the relatively older patient population evaluated is not significantly impacted by their desire to receive vasectomies.

The surveyed students, representing a significantly younger group, did show a significant increase in vasectomy interest based on sex. Most males in the study reported that they never had any interest or had a change in interest in vasectomies compared to a year ago (88.7%). Only 9.1% showed an increased interest since the overturn. In contrast, 37.1% of females had a statistically significant increase in their interest since the overturn (p = 0.001). A possible explanation for this gender-related difference in interest is the current nature of restrictive abortion access in Florida, and elsewhere, since the overturn of Roe v. Wade. On this basis, it makes sense that more females would be interested in their male partners receiving a vasectomy procedure. In a similarly conducted study of midwestern college students, surveys were sent out assessing attitudes on male-directed contraception (MDC). Average results showed female students significantly favoring MDC compared to their male counterparts. Interestingly, this study did not mention the recent overturn as a possible reason for these differences, but rather focused on societal norms [11]. Also, according to Mock et al., tubal ligation is three times more prevalent within the United States compared to vasectomies, even though it is a more complex and invasive procedure [12]. With more female students in this study showing an increased interest in their partners or future male partners receiving vasectomies, it is the responsibility of medical professionals to explain the advantages of vasectomies versus tubal ligation in terms of time, anesthesia, and surgical complications to their patients. This information can then be used to decide in favor of a vasectomy. Based on the results of this study, it is important for urologists and primary care physicians to consider talking about vasectomy procedures with not only their male patients but their female patients, as well.

Regarding knowledge, patients mostly reported knowing essentially nothing (40%) about the vasectomy procedure and the risks associated with it. It is therefore important to make sure patients who are interested in receiving a vasectomy have meaningful conversations with their urologists to be sure they are the most comfortable they can be before receiving their procedure. Meanwhile, the medical students reported knowing at least a little information (71.7%) or a lot of information (18.9%), compared to only 9.4% who reported knowing essentially nothing. This makes sense as medical students are required to learn basic information regarding male reproductive health. These student results are likely not as generalizable to the national population as specific training is geared toward these students.

When creating a standardized educational plan for urologists, data from this study may be used to put a greater emphasis on what both patients and students believe to be the most important information pieces. All 11 pre-operative topics listed by the American Urological Association should be discussed [5]. However, spending extra time on what patients believe to be the most important may help ease any patient anxiety regarding the procedure and strengthen the patient-physician relationship.

The most important piece of information to receive on vasectomy procedures that both groups wanted to know was the overall risks of the procedure and their likelihood. This is extremely important as most patients reported knowing essentially nothing about this topic. The most immediate risks associated with the procedure are relatively minor, and they include pain, hematomas, and infection [13]. Lamoury et al. also express the possibility of spontaneous recanalization and chronic scrotal pain. The most common rare complications to be reported are urethrovasocutaneous and vasocutaneous fistulas [14]. Vasectomy reversal information should be emphasized to patients next. Most patients did not know the correct reversal rate of a vasectomy procedure. They mostly guessed the reversal rate to be between 0% and 10%, while only 19% guessed the reversal rate to be between 90% and 100%. Most students guessed the reversal rate to be between 70% and 100%. According to a systemic review by Duijn et al., there was a postoperative mean patency of 81.9% (macrosurgical vasovasostomy), 90.1% (microsurgical vasovasostomy), and 92.7% (microsurgical vasovasostomy with robotic assistance) [15]. This demonstrates that most medical students had an idea of a typical vasectomy reversal, whereas patients, by majority, did not. Finally, this information should be followed by recovery time and surgical details (most patients reported knowing essentially nothing about the procedure, whereas most medical students reported having little knowledge of the procedure). Regarding recovery, patients should avoid strenuous activity, use ice packs, and not ejaculate for a week post-procedure [16]. Surgical procedural details should be discussed based on the provider technique. This information could be more extensively verbally discussed with patients, or it could be highlighted on an information sheet or pamphlet by the urology office (both physically and digitally). Specific information based on varying providers may differ based on what they consider to be the most important for their individual patients, while still aligning with the American Urological Association guidelines.

Based on this study’s results, it is recommended repeat studies be conducted to further analyze this topic. Patients and student groups had a relatively small sample size with 76 patients and 106 students, respectively. A study with a higher geographic distribution, a wider variety of patients not just associated with urology offices, and the general population not just focusing on medical students would also create higher generalizability for the results. Last, while knowledge of the reversal rate was questioned, it may be beneficial to assess knowledge of pregnancy chance after a vasectomy reversal. Patients may believe this to be the same thing, and teaching this to patients may serve to be of great value. Duijn et al. found microsurgery to be associated with the highest rate of pregnancy, with rates as high as 57.3% (less than or equal to seven years post-vasovasostomy) and 40% (greater than seven years post-vasovasostomy) [8]. This, however, is still not as high as the mean postoperative patency of microsurgical vasovasostomy (90.1%) [8]. Therefore, it may be beneficial to analyze the current population's knowledge of the difference between these values and ensure urologists teach patients about these differences as necessary.

## Conclusions

Female students showed a significantly higher interest in having their current or possible future partner undergo a vasectomy procedure compared to their male counterparts and compared to mostly older patients within urology clinics. This may be associated with the recent change in abortion laws following the overturn of Roe v. Wade. It is therefore important that urologists, primary care physicians, gynecologists, etc., remember to counsel interested female patients about vasectomy procedures, as more may now be interested in learning about it since abortion laws have changed across the country. An educational plan should be created for patients interested in receiving a vasectomy highlighting what patients and students ranked as the most important information. Urologists should cover the American Urological Association guidelines and may find use in placing emphasis on the overall risks and their likelihood, the likelihood of reversal, and the recovery time and surgical details. This may help ease more patients’ anxiety with the procedure and strengthen the patient-physician relationship.
